# *Gardnerella* enrichment in the vaginal microbiome of women with gestational diabetes mellitus is associated with lower fetal birthweight percentiles

**DOI:** 10.1007/s00125-026-06770-x

**Published:** 2026-06-11

**Authors:** Yilin Fu, Nary Long, Phannaroat Sourn, Mengqi Zhang, Weidong Tan, Huihui Yu, Junjie Yuan, Yuxin Chen, Jing Wang, Xue Zhang, Xin Li, Shaoshuai Wang, Ling Feng, Jianli Wu, Zizhuo Wang, Wencheng Ding

**Affiliations:** 1 Department of Obstetrics and Gynecology, Tongji Hospital, Tongji Medical College, Huazhong University of Science and Technology, Wuhan, Hubei China; 2 National Clinical Research Center for Obstetrics and Gynecology, Tongji Hospital, Tongji Medical College, Huazhong University of Science and Technology, Wuhan, China

**Keywords:** Fetal growth, *Gardnerella*, Gestational diabetes mellitus, Pregnancy, Vaginal microbiome

## Abstract

**Aims/hypothesis:**

We aimed to characterise alterations in the late-pregnancy vaginal microbiota in women with gestational diabetes mellitus (GDM), and to examine their associations with maternal glycaemic status and fetal growth.

**Methods:**

In this observational case–control study, women with newly diagnosed, diet-managed GDM (*n*=60) and healthy pregnant control participants (*n*=119) were recruited at a single tertiary centre. Vaginal swabs were collected in the third trimester and analysed by full-length 16S rRNA gene sequencing. Associations between vaginal microbiota composition, maternal glycaemic measures, and fetal birthweight percentile (FBW%ile) were evaluated.

**Results:**

The overall vaginal microbial composition differed between groups despite similar alpha diversity. Beta diversity analyses showed significant separation between the women with GDM and the control group (*R*=0.068, *p*=0.011 by ANOSIM). *Lactobacillus-*dominated communities were less frequent in women with GDM than in control participants (73.3% vs 86.6%, *p*=0.029), with reduced *Lactobacillus* abundance (79.8% vs 87.4%, nominal *p*=0.020) and increased *Gardnerella* abundance (9.7% vs 1.4%, nominal *p*=0.011). Within the GDM group, *Gardnerella* abundance correlated positively with fasting glucose levels (*r*=0.3617, nominal *p*=0.007) and inversely with FBW%ile (*r*=−0.2774, nominal *p*=0.032). Stratification by FBW%ile (<50% vs ≥50%) showed a higher proportion of non-*Lactobacillus-*dominated communities (44.4% vs 12.1%, *p*=0.001) and higher *Gardnerella* abundance (15.0% vs 3.2%, nominal *p*=0.020) in the low FBW%ile subgroup. Associations with *Gardnerella* were attenuated in sensitivity analyses that excluded samples with extreme *Gardnerella* abundance.

**Conclusions/interpretation:**

Late-pregnancy GDM was associated with modest shifts in vaginal microbial structure, characterised by reduced *Lactobacillus* dominance and relative enrichment of *Gardnerella*. Exploratory analyses of associations of *Gardnerella* with maternal fasting glucose levels and fetal growth suggest that variation in the vaginal microbial environment may contribute to metabolic and fetal growth heterogeneity in women with GDM.

**Graphical Abstract:**

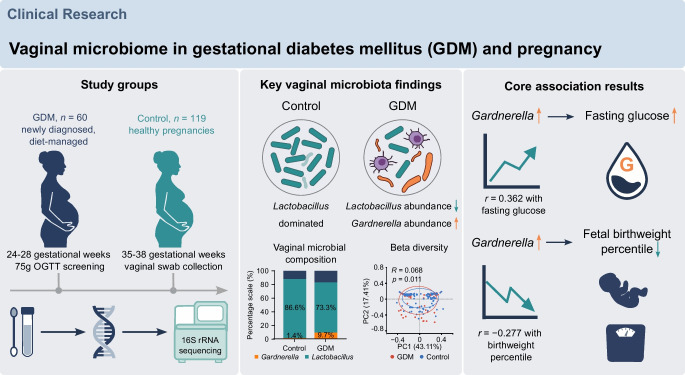

**Supplementary Information:**

The online version of this article (10.1007/s00125-026-06770-x) contains peer-reviewed but unedited supplementary material.


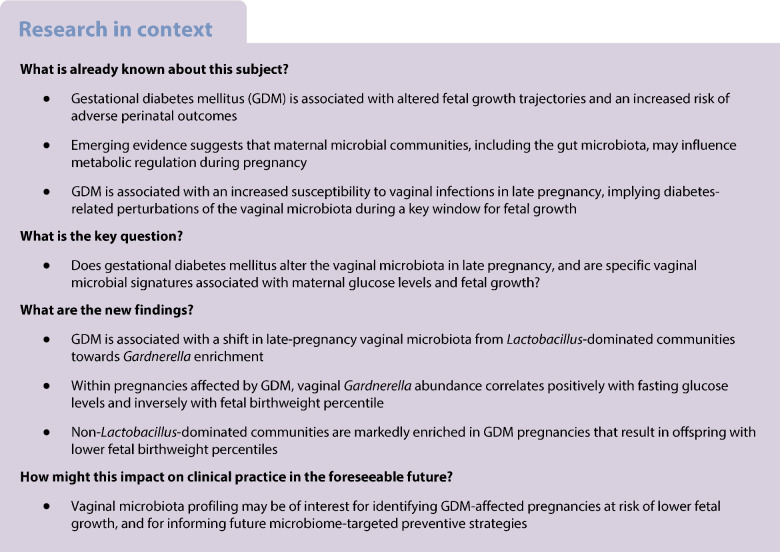


## Introduction

A healthy vaginal microbiota is typically dominated by *Lactobacillus*, which maintains an acidic environment and protects against genital tract infections [1]. Disruption of this equilibrium, termed vaginal dysbiosis, is characterised by depletion of *Lactobacillus* and overgrowth of anaerobic bacteria. Such dysbiosis has been consistently associated with adverse pregnancy outcomes, including preterm birth, premature rupture of the membranes, and intrauterine infection [2, 3]. However, the microbial signatures and functional implications of vaginal dysbiosis across specific pregnancy complications remain incompletely defined, underscoring the need for more nuanced characterisation of vaginal microbial alterations during pregnancy.

Gestational diabetes mellitus (GDM), defined as glucose intolerance with onset or first recognition during pregnancy, affects approximately 14% of pregnancies worldwide and represents a major public health concern [4, 5]. Growing evidence suggests that microbial dysbiosis across maternal niches, particularly the gut, may contribute to insulin resistance, chronic low-grade inflammation and metabolic dysfunction in women with GDM [6–9]. In parallel, associations between GDM and vaginal infections, including bacterial vaginosis (BV) and vulvovaginal candidiasis (VVC), are well documented [10–12]. However, systematic investigations of the vaginal microbiota in women with GDM remain limited. Most studies rely on Nugent scoring or culture-based approaches, which provide limited taxonomic resolution and fail to capture the complexity of vaginal microbial communities [12–15]. Accordingly, evidence for vaginal microbiota alterations in women with GDM remains sparse and inconsistent across cohorts and methodologies [13, 16–18], highlighting the need for culture-independent, high-resolution microbiome profiling in this population.

GDM is well recognised for its impact on fetal growth and the long-term health of the offspring [19]. Maternal hyperglycaemia drives fetal hyperinsulinaemia, which is the primary mechanism underlying the excess risk of macrosomia, the hallmark adverse fetal outcome of GDM [4, 20]. Interestingly, beyond this well-established accelerated fetal growth phenotype, maternal microbial environments may further influence fetal growth trajectories, potentially through microbiome-mediated modulation of maternal metabolism, inflammation and endocrine signalling [21]. Notably, pregnancies affected by GDM that are complicated by vaginal bacterial infections have also been shown to be associated with increased risk of low birthweight, suggesting that disturbances of the vaginal microbial ecosystem may contribute to divergent fetal growth outcomes [12]. Recent data further indicate that fetal sex may modify susceptibility to adverse growth patterns for women with GDM, with male fetuses showing greater vulnerability to both macrosomia and, paradoxically, being small for gestational age (SGA) in some cohorts [22, 23]. Whether alterations in the maternal vaginal microbiota contribute to fetal growth regulation in women with GDM, and whether such associations are modified by fetal sex, remains largely unexplored.

Taken together, these data highlight a key knowledge gap regarding the composition and potential metabolic relevance of the vaginal microbiota in women with GDM, particularly in relation to maternal glycaemic traits and fetal growth heterogeneity. To address this, we conducted a prospective cohort study using full-length 16S rRNA gene sequencing to characterise the vaginal microbiota of pregnant women with GDM. Specifically, we aimed to identify GDM-associated alterations in vaginal microbial composition and structure, examine their relationships with maternal glycaemic traits, and investigate associations with fetal growth patterns, with particular emphasis on sex-specific differences. Elucidating these relationships is essential for advancing the understanding of how vaginal microbial ecosystems may contribute to metabolic heterogeneity in women with GDM and variability in fetal growth outcomes.

## Methods

### Study design and participants

This observational case–control study was conducted at Tongji Hospital, Tongji Medical College, Huazhong University of Science and Technology, China, between March 2024 and March 2025. Singleton pregnancies were recruited during routine 75 g oral glucose tolerance test (OGTT) screening at 24–28 weeks’ gestation, in accordance with International Association of Diabetes and Pregnancy Study Groups (IADPSG) criteria [24]. GDM was diagnosed using fasting blood glucose (FBG), 1 h and 2 h plasma glucose thresholds of 5.1, 10.0 and 8.5 mmol/l, respectively; one or more values meeting or exceeding these thresholds was considered diagnostic. Only women with GDM that was managed through diet alone were included, representing mild GDM. Gestational age was determined based on the first day of the last menstrual period, and confirmed or corrected by first-trimester ultrasonography when available. Healthy pregnant women with singleton gestations served as the control group.

Participants were recruited from the obstetrics outpatient clinics of a tertiary referral centre in Wuhan, central China. The majority of participants were Han Chinese, reflecting the local population structure. The study population is broadly representative of pregnant women receiving care in this region, although it may not reflect populations in other regions or with different ethnic compositions. Information on socioeconomic status and educational level was not available. Race and ethnicity information was derived from the routine electronic medical record system.

Women with GDM were eligible for inclusion only in the absence of other medical or obstetric comorbidities. Exclusion criteria were hypertensive disorders of pregnancy, prior hyperglycaemia or pre-existing diabetes, thyroid dysfunction, hepatitis B virus infection, group B *Streptococcus* colonisation, VVC, systemic lupus erythematosus or other connective tissue diseases. In addition, women who had used systemic or intravaginal antibiotics or antifungal agents within 2 weeks before sampling were also excluded. To minimise transient perturbations of the vaginal microbiota, women were further excluded if they had engaged in sexual intercourse or performed vaginal douching within 72 h before sampling.

Cases were defined as pregnant women with a first diagnosis of GDM at routine screening at 24–28 weeks who were managed with diet alone. Control participants were healthy pregnant women without GDM. To minimise confounding by pregnancy stage, control participants were matched to cases by gestational age at sampling (GAS) within a predefined range of ±2 weeks. Approximately two control participants were included per case where possible. GAS was therefore comparable between groups by design.

Maternal and neonatal clinical data were retrieved from the electronic medical record system of Tongji Hospital. Fetal birthweight percentile (FBW%ile) and fetal birth head circumference percentile (FBHC%ile) were calculated according to the INTERGROWTH-21st standards [25]. The study was approved by the Medical Ethics Committee of Tongji Hospital, Tongji Medical College (TJ-IRB202412216), and performed in accordance with the Declaration of Helsinki. All participants provided written informed consent prior to enrolment for publication of the data and the results obtained.

### Vaginal sample collection

All vaginal swab samples collected during the third trimester (35–38 gestational weeks) were obtained by a trained clinician in accordance with a standardised operating procedure. Briefly, a sterile single-use flocked swab (BKMAM Biotech., Changde, China) was gently inserted into the posterior vaginal fornix under direct visualisation using a sterile speculum, avoiding contact with the external genitalia. The swab was rotated for approximately 10 s to ensure adequate sampling, immediately placed into a sterile cryovial, and stored at −80°C until further processing.

### DNA extraction, amplification and sequencing

Microbial genomic DNA was extracted from vaginal swab samples using FastPure Stool DNA isolation kits (MJYH, Shanghai, China) according to the manufacturer’s instructions. DNA concentration and purity were assessed using a NanoDrop 2000 spectrophotometer (Thermo Fisher Scientific), and DNA integrity was evaluated by 1% agarose gel electrophoresis.

The full-length bacterial 16S rRNA gene was amplified using the barcoded universal primers 27F (5′-AGRGTTYGATYMTGGCTCAG-3′) and 1492R (5′-RGYTACCTTGTTACGACTT-3′) [26]. PCR amplification was performed in a 20 μl reaction system containing 4 μl 5× FastPfu buffer, 2 μl 2.5 mmol/l dNTPs, 0.8 μl of each primer (5 μmol/l), 0.4 μl FastPfu DNA polymerase, 10 ng of template DNA, and nuclease-free water up to a volume of 20 μl. Amplifications were carried out on an T100 thermal cycler (Bio-Rad, Hercules, CA, USA) under the following conditions: initial denaturation at 95°C for 3 min, followed by 27 amplification cycles consisting of denaturation at 95°C for 30 s, annealing at 60°C for 30 s, and extension at 72°C for 45 s, with a final extension at 72°C for 10 min. To minimise amplification bias, all samples were amplified using the same cycling conditions.

PCR products were verified by 2% agarose gel electrophoresis, purified using a PCR Clean-Up Kit (YuHua, Shanghai, China), and quantified using a Synergy HTX microplate reader (BioTek, Winooski, VT, USA). Purified amplicons were pooled at equimolar concentrations. SMRTbell libraries were constructed using a SMRTbell prep kit 3.0 (Pacific Biosciences, Menlo Park, CA, USA) and sequenced on the PacBio Revio system (Pacific Biosciences, Menlo Park, CA, USA) by Majorbio Bio-Pharm Technology (Shanghai, China). Circular consensus sequencing was performed using SMRT Link software version 11.0 to generate high-fidelity reads.

### OTU-based sequence processing and taxonomic annotation

High-fidelity reads were de-multiplexed based on sample-specific barcodes and filtered by length (1000–1800 bp). Quality-controlled sequences were de-replicated, and chimeric sequences were identified and removed using the UCHIME algorithm implemented in the UPARSE pipeline (USEARCH/UPARSE version 11). The remaining high-quality sequences were clustered into operational taxonomic units (OTUs) at 97% sequence similarity using UPARSE version 11. Representative sequences for each OTU were selected for downstream taxonomic assignment, and sequences annotated as chloroplasts or mitochondria were excluded.

To minimise the influence of uneven sequencing depth on downstream analyses, the OTU table was rarefied to 20,368 sequences per sample, at which point the mean Good’s coverage across samples exceeded 99%. Taxonomic classification of representative OTU sequences (with a confidence threshold of 70%) was performed using the RDP classifier (version 2.2) against a custom 16S rRNA gene database (NT_Taxon_core_v2024/16s_bacteria) that incorporates the NCBI core nucleotide database (version 2024) and the NCBI 16S rRNA database (https://ftp.ncbi.nlm.nih.gov/blast/db/).

### Bioinformatic analysis

The Majorbio Cloud Platform (https://cloud.majorbio.com) was used to process full-length 16S rRNA sequencing data generated by Majorbio Bio-Pharm Technology. Alpha diversity indices, including Chao1, Shannon and Simpson indices, were calculated using Mothur software (version 1.30.2). Group differences in alpha diversity were assessed using the Wilcoxon rank-sum test. Beta diversity was evaluated based on the Bray–Curtis or UniFrac distance, and visualised by principal coordinate analysis (PCoA) and non-metric multidimensional scaling, using analysis of similarities (ANOSIM) to determine statistical significance. Differential abundant analysis was performed using MaAsLin3 version 1.2.0 (available at https://huttenhower.sph.harvard.edu/maaslin) [27], with group as the fixed effect and including age and GAS as covariates. Spearman correlation analysis was used to assess associations between microbial taxa and clinical variables. Multiple testing was corrected using the Benjamini–Hochberg false discovery rate (FDR) method, where *q*<0.20 was considered nominally suggestive, while formal statistical significance was defined as *q*<0.05. The PICRUSt2 tool combined with the KEGG and the COG database was used to predict biological functions (https://github.com/picrust/picrust2) [28]. Linear regression analyses were additionally performed. Effect modification by fetal sex was evaluated using interaction testing, and by comparing nested models with and without the interaction term.

#### Statistical analysis

Statistical analyses were performed using IBM SPSS Statistics for Windows (version 27.0, IBM, Armonk, NY, USA), GraphPad Prism (version 10.0.0, GraphPad Software, San Diego, CA, USA) and R software (version 4.5.1, R Foundation for Statistical Computing, Vienna, Austria). Continuous variables were assessed for normality, and are presented as mean ± SD or median (IQR), as appropriate. Normally distributed variables were compared using two-tailed Student’s *t* tests, while non-normally distributed variables were analysed using Wilcoxon rank-sum tests. Categorical variables were compared using the χ^2^ test. All statistical tests were two-sided, and *p*<0.05 was considered statistically significant.

## Results

### Study population

**Fig. 1:**
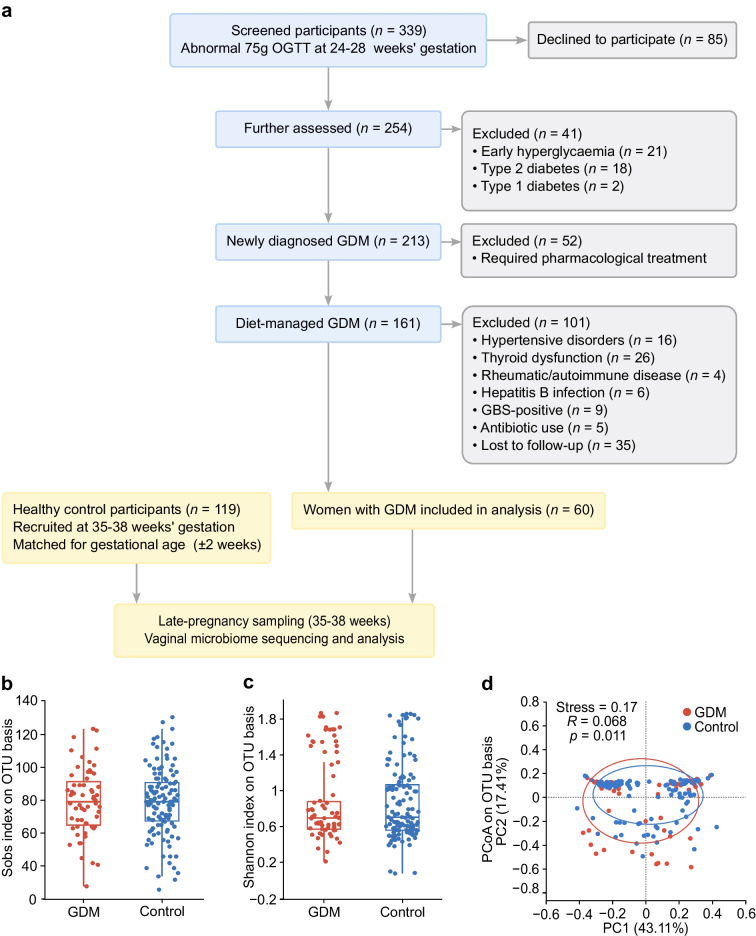
Participant flow chart and vaginal microbiota diversity in late pregnancy in women with GDM and healthy control participants. (**a**) Flow chart of participant recruitment. A total of 60 women with newly diagnosed, diet-managed GDM and 119 healthy pregnant control participants were included in the final analysis. Control participants were matched to cases on the basis of GAS to within ±2 weeks. (**b**, **c**) Comparison of vaginal microbiota richness (Sobs index) and diversity (Shannon index) on OTU basis between GDM and control groups, showing no significant difference between groups. (**d**) PCoA on OTU basis, based on Bray–Curtis distances, demonstrating a significant difference in vaginal microbiota community composition between the GDM and control groups (ANOSIM: *R*=0.068, *p*=0.011), with an acceptable ordination stress value (stress=0.17). GBS, group B *Streptococcus*

A total of 339 women with singleton pregnancies and abnormal 75 g OGTT results at 24–28 weeks’ gestation were initially screened for the GDM group. After 85 declined to participate, 254 underwent further assessment. Women with hyperglycaemia earlier in pregnancy or pre-existing diabetes (*n*=41) were excluded, leaving 213 women with newly diagnosed GDM. Of these, 52 who subsequently required pharmacological treatment were excluded. After further exclusion of women with pregnancy-related comorbidities or complications *(n*=66) and loss to follow-up before late-pregnancy sampling (*n*=35), the GDM group comprised 60 women with newly diagnosed GDM managed exclusively with dietary intervention. The control group included 119 healthy pregnant women recruited at 35–38 weeks’ gestation and matched for GAS (±2 weeks). The participant flow chart is shown in Fig. 1a. Compared with control participants, women with GDM had higher glucose measurements, but other clinical and obstetric variables did not differ significantly between groups (Table 1).

**Table 1:** Characteristics and pregnancy outcomes for women with GDM and the control group

Characteristics and outcomes	Control group (*n*=119)	GDM group (*n*=60)	*p* value
Maternal age (years)	30.0 (28.0–32.0)	31.0 (29.0–33.8)	0.059^a^
Pre-pregnancy BMI (kg/m^2^)	20.8 (19.5–22.6)	21.0 (19.4–23.6)	0.404^a^
Gravidity			0.091^b^
1	91 (76.5)	41 (68.3)	
2	25 (21.0)	13 (21.7)	
>2	3 (2.5)	6 (10.0)	
Gestational age at sampling (weeks)	36.4 (36.0–37.1)	36.6 (36.0–37.1)	0.962^a^
FBG	4.4 ± 0.3	4.8 ± 0.5	<0.001^c^
1 h blood glucose	7.8 (6.5–8.7)	10.3 (9.3–11.3)	<0.001^a^
2 h blood glucose	6.5 (5.8–7.2)	8.9 (7.9–9.8)	<0.001^a^
Gestational age at delivery (weeks)	39.1 ± 0.9	38.9 ± 0.8	0.100^c^
Caesarean delivery	71 (59.7)	38 (63.3)	0.635^b^
Birthweight (g)	3110 (2930–3400)	3105 (2993–3387)	0.626^a^
FBW%ile	43.9 (24.7–69.2)	43.7 (33.4–73.0)	0.195^a^
FBHC%ile	69.1 (35.2–90.6)	73.5 (47.3–88.5)	0.365^a^
Fetal gender			0.955^b^
Male	64 (53.8)	32 (53.3)	
Female	55 (46.2)	28 (46.7)	
Apgar score at 1 min	8.0 (8.0–8.0)	8.0 (8.0–8.0)	0.621^a^
Apgar score at 5 min	9.0 (9.0–9.0)	9.0 (9.0–9.0)	1.000^a^
SGA	10 (8.4)	3 (5.0)	0.408^b^
LGA	10 (8.4)	5 (8.3)	0.987^b^

Values for continuous variables are presented as means ± SD or medians (IQR); values for categorical variables are expressed as *n* (%)

^a^Using the Wilcoxon rank-sum test

^b^Using the χ^2^ test

^c^Using Student's *t* tests

After removal of contaminant sequences and rarefaction, samples were normalised to 20,368 high-quality reads per sample. Across all samples, 471 bacterial OTUs were identified. Core and pan-microbiota accumulation curves were comparable between the GDM and control groups and approached saturation, consistent with adequate sequencing depth for downstream community profiling (ESM Fig. 1a, b).

### Vaginal microbial diversity patterns in the GDM and control groups

The rarefaction curves for richness and diversity metrics (Sobs and Shannon indices) plateaued across samples, indicating adequate sequencing depth (ESM Fig. 1c, d). No significant between-group differences were detected for alpha diversity (Sobs and Shannon indices; Fig. 1b, c). In contrast, non-metric multidimensional scaling indicated that beta diversity differed between the GDM and control groups (*R*=0.068, *p*=0.011) with acceptable ordination fit (stress=0.17) (Fig. 1d).

### Taxonomic shifts and community-type distributions in women with GDM

**Fig. 2:**
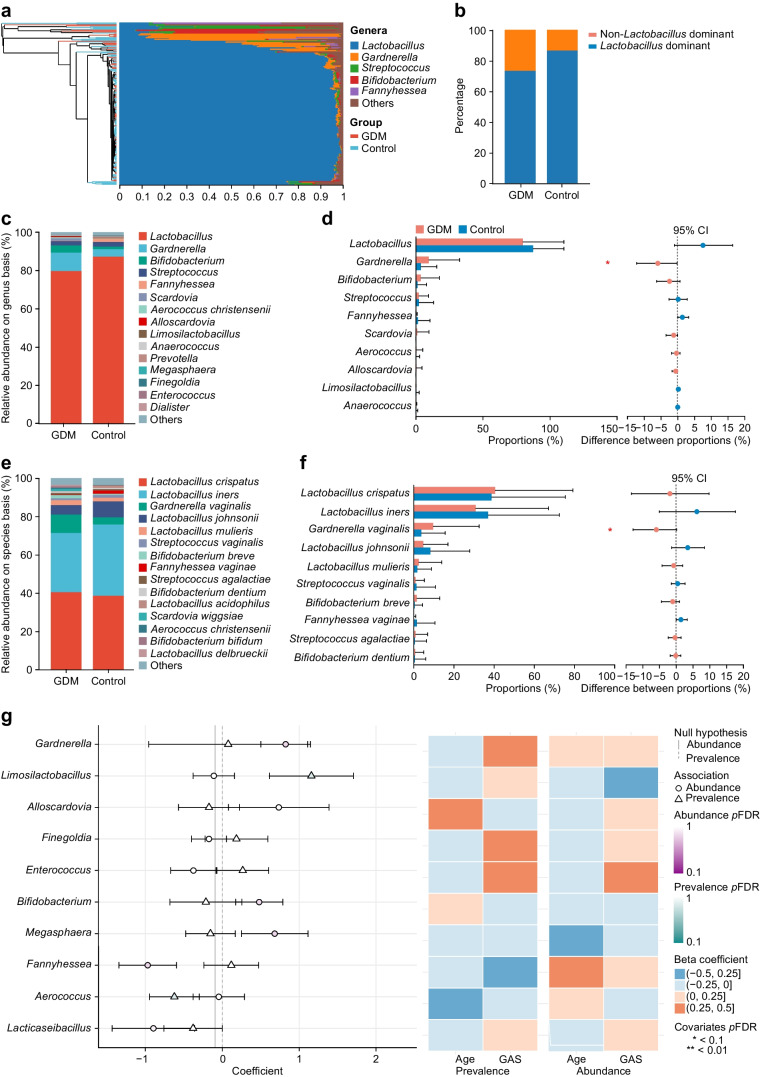
Taxonomic shifts and vaginal microbiota in late pregnancy in women with GDM and control participants. (**a**) Hierarchical clustering of genus-level vaginal microbiota profiles across all participants, showing relative abundances of the dominant genera shown for individual samples. Samples are annotated by study group (GDM or control). (**b**) Proportions of *Lactobacillus-*dominated communities in the GDM and control groups (73.3% vs 86.6%, *p*=0.029, χ^2^ test). (**c**, **d**) Genus-level composition of the vaginal microbiota in the two groups. The stacked bar plots show the relative abundances of the top 10 genera, and group comparisons are shown as mean proportions, differences between proportions and 95% CI. The Wilcoxon rank-sum test was used for group comparisons. For multiple comparisons, the Benjamini–Hochberg method was applied, with FDR<0.20 considered nominally suggestive; *p<0.05 based on the Wilcoxon rank-sum test. (**e**, **f**) Species-level composition of the vaginal microbiota in the two groups. The stacked bar plots show the relative abundances of the top 10 species, and group comparisons are shown as mean proportions, differences between proportions and 95% CI. The Wilcoxon rank-sum test was used for group comparisons. FDR<0.20; *p<0.05 based on the Wilcoxon rank-sum test. (**g**) Differential abundance and prevalence analysis of selected genera using MaAsLin3, with study group as the fixed effect and maternal age and GAS as covariates. The error bars represent SEM, circles indicate abundance coefficients, and triangles indicate prevalence coefficients. Heatmaps show beta coefficients and *q* values (FDR-corrected) for covariates: *q<0.10; **q<0.01 (after FDR correction, no association between any covariate and genus reached *q*<0.10 so no asterisks shown on figure)

At the genus level, the vaginal microbiota was dominated by *Lactobacillus*, *Gardnerella*, *Streptococcus*, *Bifidobacterium* and *Fannyhessea* (Fig. 2a). The proportion of *Lactobacillus*-dominated samples was lower in women with GDM than in control participants (73.3% vs 86.6%, *p*=0.029) (Fig. 2b). The relative abundance of *Lactobacillus* was lower in women with GDM (79.8% vs 87.4%, *q*=0.102, nominal *p*=0.020), whereas that for *Gardnerella* was higher (9.7% vs 1.4%, *q*=0.102, nominal *p*=0.011) (Fig. 2c, d). At the species level, the relative abundance of *Lactobacillus iners* was lower in women with GDM (30.9% vs 37.1%, *q*=0.197, nominal *p*=0.039), whereas that for *Gardnerella vaginalis* was higher (9.7% vs 1.4%, *q*=0.108, nominal *p*=0.011) (Fig. 2e, f). After adjusting for maternal age and GAS using MaAsLin3, the difference in *Lactobacillus* abundance between groups was no longer significant (β=−0.20; 95% CI −0.52, 0.12; *q*=1.000, nominal *p*=0.658), whereas *Gardnerella* abundance remained directionally higher in women with GDM compared with control participants (β=0.82; 95% CI 0.19, 1.45; *q*=0.666, nominal *p*=0.016) (Fig. 2g).

### Associations between vaginal taxa and FBW%ile in women with GDM

**Fig. 3:**
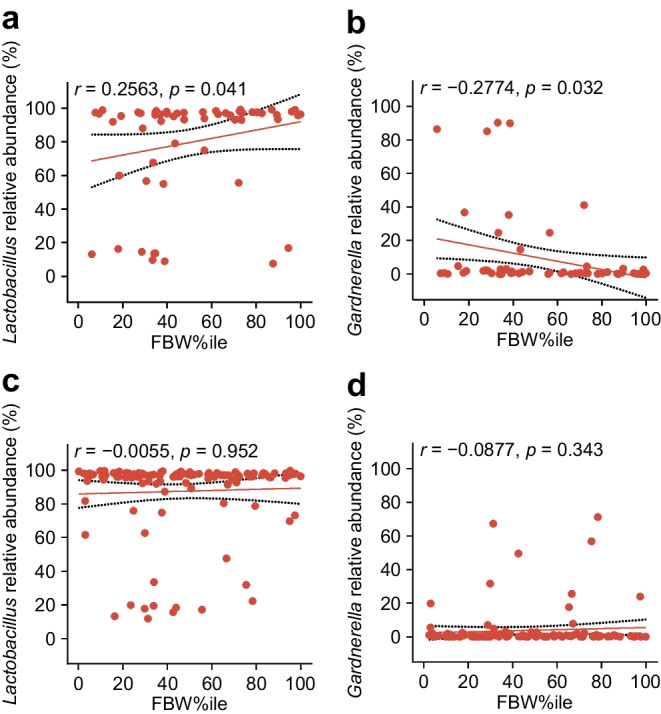
Associations between vaginal bacterial relative abundance and FBW%ile. (**a**, **b**) Correlations between FBW%ile and the relative abundance of *Lactobacillus* and *Gardnerella* in the GDM group. (**c**, **d**) Correlations between FBW%ile and the relative abundance of *Lactobacillus* and *Gardnerella* in the control group. Solid lines indicate fitted regression lines, and dotted lines indicate 95% CI

Within the GDM group, Spearman correlation analyses were performed to assess associations between microbial taxa and clinical variables. After FDR correction, no correlations reached significance (ESM Fig. 2a, b). At the genus level, *Lactobacillus* abundance showed a positive correlation with FBW%ile (*r*=0.2563, nominal *p*=0.041) (Fig. 3a), whereas *Gardnerella* correlated negatively with FBW%ile (*r*=−0.2774, nominal *p*=0.032) (Fig. 3b). Performing a sensitivity analysis that excluded four samples with extreme *Gardnerella* abundance (>80%) attenuated the correlation (*r*=−0.18; 95% CI −0.42, 0.09; *p*=0.17) (ESM Fig. 2c), showing the substantial influence of these outliers, although the direction remained negative. In control participants, no significant or nominal correlations were observed between *Lactobacillus* or *Gardnerella* and fetal size metrics (Fig. 3c, d).

### Associations between vaginal taxa and OGTT glucose values in women with GDM

**Fig. 4:**
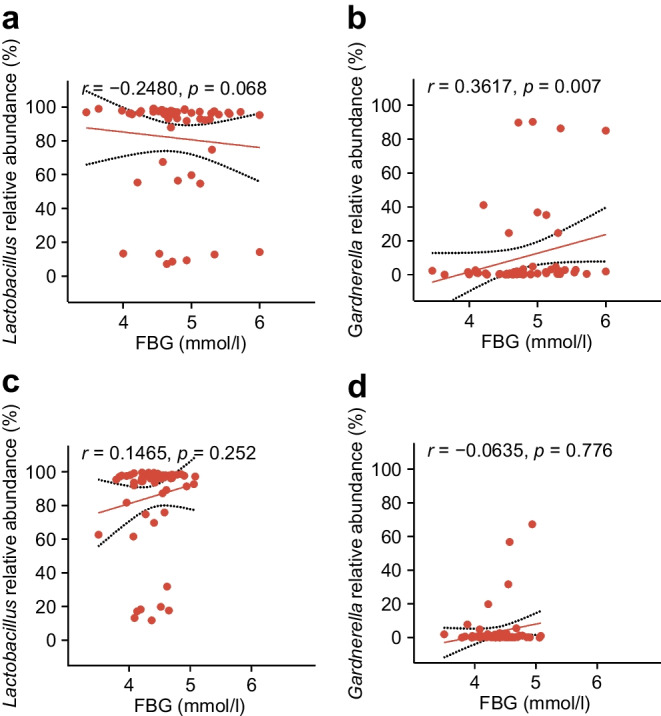
Correlations between vaginal bacterial relative abundance and FBG. (**a**, **b**) Correlations between FBG and the relative abundance of *Lactobacillus* and *Gardnerella* in the GDM group. (**c**, **d**) Correlations between FBG and the relative abundance of *Lactobacillus* and *Gardnerella* in the control group. Solid lines indicate fitted regression lines, and dotted lines indicate 95% CI

We next examined associations between blood glucose and the vaginal microbiota in women with GDM. The vaginal microbiota composition correlated with OGTT glucose measurements (ESM Fig. 2d). *Lactobacillus* showed a non-significant negative trend (*r*=−0.2480, *q*=0.767, nominal *p*=0.068) (Fig. 4a), whereas *Gardnerella* was positively correlated with the FBG value (*r*=0.3617, *q*=0.200, nominal *p*=0.007) (Fig. 4b). No significant or nominal correlations were detected between *Lactobacillus* or *Gardnerella* and 1 h or 2 h blood glucose values (ESM Fig. 2d). In control participants, corresponding analyses identified no significant or nominal correlations between these taxa and OGTT glucose measurements (Fig. 4c, d). Exclusion of four samples with extreme *Gardnerella* abundance (>80%) attenuated the correlation with FBG, although it remained significant (*r*=0.296; 95% CI 0.014, 0.535; *p*=0.035) (ESM Fig. 2e).

### Fetal sex-stratified correlations in women with GDM

To assess potential sex-specific effects, analyses were stratified by fetal sex. Baseline maternal characteristics were similar between strata, but FBW%ile was higher in male fetuses (ESM Table 1). In male fetuses, *Gardnerella* abundance was inversely correlated with FBW%ile (*r*=−0.4573, *p*=0.009) and positively correlated with FBG (*r*=0.4413, *p*=0.015) (ESM Fig. 3a, b). These correlations were not significant in female fetuses (ESM Fig. 3c, d). In regression analyses, the association of *Gardnerella* with FBW%ile and FBG was stronger in male fetuses than in female fetuses. However, the confidence intervals overlapped, and no statistically significant interaction by fetal sex was detected (*p*_interaction_=0.126 for FBW%ile; *p*_interaction_=0.235 for FBG) (ESM Fig. 3e, f).

### Vaginal microbiota features in GDM-affected pregnancies stratified by FBW%ile

**Fig. 5:**
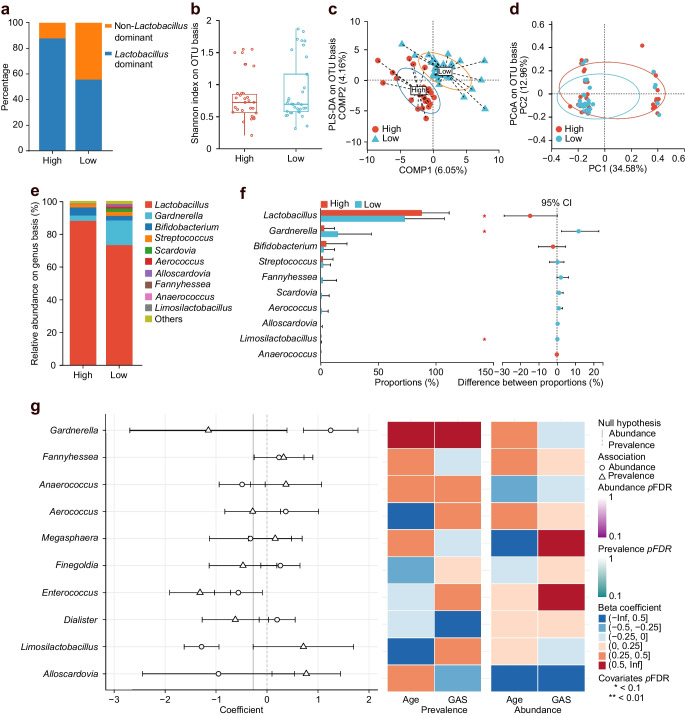
Low FBW%ile for women with GDM is associated with *Gardnerella* enrichment in the vaginal microbiome. Participants were categorised into low FBW%ile (<50%) and high FBW%ile (≥50%). (**a**) Proportions of *Lactobacillus-*dominated communities in the high and low FBW%ile groups (87.9% vs 55.6%, *p*=0.005, χ^2^ test). (**b**) Comparison of vaginal microbiota alpha diversity (Shannon index) between high and low FBW%ile groups on OTU basis. (**c**) Partial least squares discriminant analysis (PLS-DA) demonstrating separation of vaginal microbial communities between the high and low FBW%ile groups. (**d**) PCoA based on unweighted UniFrac distance showing significant differences in community structure; group differences were evaluated by ANOSIM. (**e**) Relative abundance of the top 10 genera. (**f**) Comparison of the top 10 genera between groups, with mean proportions, differences between proportions and 95% CI. FDR<0.20; *p<0.05 based on the Wilcoxon rank-sum test. (**g**) Differential abundance and prevalence analysis of selected genera using MaAsLin3, with subgroup as the fixed effect and maternal age and GAS as covariates. The error bars represent SEM, circles indicate abundance coefficients, and triangles indicate prevalence coefficients. Heatmaps show beta coefficients and *q* values (FDR-corrected) for covariates: *q<0.10; **q<0.01 (after FDR correction, no association between any covariate and genus reached *q*<0.10 so no asterisks shown on figure). Inf, infinity

Women with GDM were categorised as having a high FBW%ile (≥50%) or a low FBW%ile (<50%). The low FBW%ile group had a higher proportion of non-*Lactobacillus-*dominated communities than the high FBW%ile group (44.4% vs 12.1%, *p*=0.001) (Fig. 5a). Alpha diversity did not differ significantly, while multivariate analyses indicated differences in community structure: partial least squares discriminant analysis showed group separation, and PCoA based on unweighted UniFrac distances supported between-group differences (*R*=0.067, *p*=0.022) (Fig. 5b–d).

Taxonomically, the low FBW%ile group exhibited lower *Lactobacillus* abundance (73.2% vs 89.7%, *q*=0.159, nominal *p*=0.039) and higher *Gardnerella* abundance (15.0% vs 3.2%, *q*=0.159, nominal *p*=0.020) (Fig. 5e, f). After adjusting for maternal age and GAS using MaAsLin3, the difference in *Lactobacillus* abundance between groups was no longer significant (β=−0.54; 95% CI −1.08, 0.01; *q*=0.963, nominal *p*=0.492), while *Gardnerella* showed a directionally higher abundance (β=1.25; 95% CI 0.20–2.30; *q*=0.836, nominal *p*=0.015) (Fig. 5g).

### Predicted functional profiles and correlation patterns

**Fig. 6:**
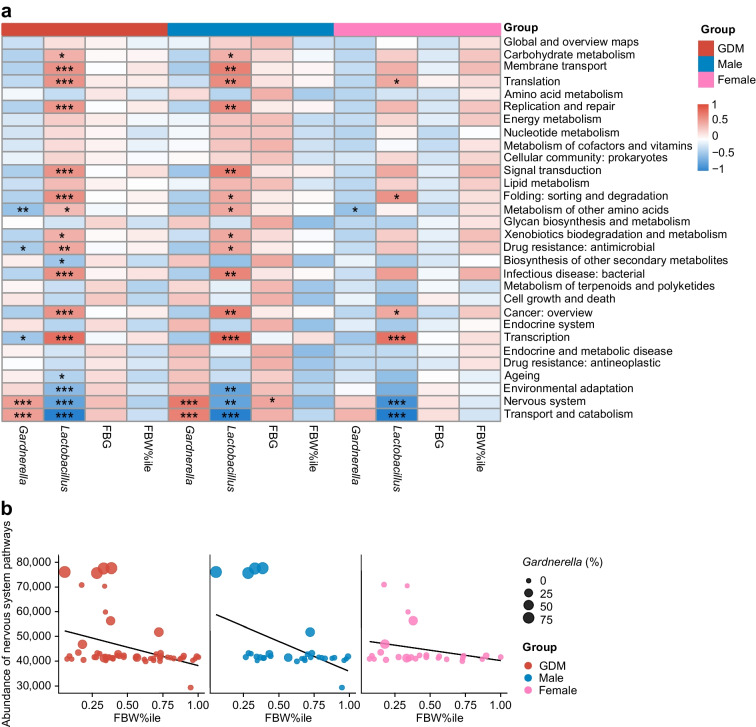
Associations between predicted vaginal microbiome functions and fetal growth, FBG and vaginal microbiota in women with GDM. (**a**) Spearman correlation heatmap showing associations between distinct vaginal microbiota (*Gardnerella* and *Lactobacillus*), maternal glycaemic measures and FBW%ile and the top 30 predicted KEGG pathways ranked by mean abundance, analysed across GDM pregnancies and stratified by fetal sex. *q<0.05; **q<0.01; ***q<0.001 (FDR-corrected). (**b**) Associations between the predicted KEGG ‘nervous system’ pathway and FBW%ile in the overall GDM group and in male and female fetuses separately, showing a stronger inverse relationship aligned with higher *Gardnerella* abundance in male fetuses

Functional profiles were inferred from 16S rRNA gene data using PICRUSt2, and are presented as predicted features rather than directly measured activities. Predicted features were predominantly assigned to metabolic and transport-related categories across COG and KEGG annotations (ESM Fig. 4a–c). Correlation analyses were performed between selected taxa, FBG, FBW%ile and the top 30 predicted pathways ranked by mean abundance, with FDR correction applied (Fig. 6a). Among the KEGG categories, the pathway group annotated as ‘nervous system’ showed correlation patterns with *Gardnerella*, FBG and FBW%ile (*q*<0.1) (Fig. 6a, b). A sensitivity analysis excluding four samples with extremely high *Gardnerella* abundance (>80%) attenuated the correlation (ESM Fig. 5a). Within this category, predicted pathways annotated as ‘GABAergic synapse’ and ‘glutamatergic synapse’ displayed similar directional associations with *Gardnerella*, FBG and FBW%ile (*q*<0.01) (ESM Fig. 5b).

## Discussion

This prospective study characterised the vaginal microbiota in late pregnancy among women with GDM, and examined its relationships with maternal glucose levels and fetal growth. GDM-affected pregnancies showed compositional shifts, including reduced *Lactobacillus* dominance and a directional increase in *Gardnerella* abundance. Within the GDM group, *Gardnerella* abundance was nominally associated with higher maternal FBG and lower FBW%ile, although these associations were attenuated after excluding extreme outliers. These findings should therefore be considered exploratory, suggesting that variation in vaginal microbial composition may contribute to heterogeneity in metabolic status and fetal growth in women with GDM.

The observed structural imbalance, which occurred without increased microbial diversity, indicates reduced *Lactobacillus* dominance with relative enrichment of taxa linked to dysbiosis, particularly *Gardnerella*. Given that late pregnancy is typically characterised by a low-diversity, *Lactobacillus*-dominated ecosystem maintained by strong host selection pressures, including hormonal regulation, glycogen availability, acidic pH and immune tolerance [29], these findings suggest that GDM is associated with a subtle ecological reconfiguration within an already highly selected niche.

As *Lactobacillus* is central to maintaining vaginal acidity and mucosal stability, its depletion may indicate reduced ecological resilience and impaired colonisation resistance [1, 30, 31]. Conversely, *Gardnerella*, which is a key genus associated with BV, has been linked to vaginal dysbiosis, local inflammation, and adverse pregnancy outcomes [32–35]. Such compositional shifts may compromise the mucosal barrier and disrupt local immune homeostasis, thereby increasing susceptibility to opportunistic infections, which is consistent with previous reports of higher rates of BV, group B *Streptococcus* colonisation and VVC in women with GDM [10, 12, 18].

Hyperglycaemia during pregnancy has consistently been shown to be associated with increased susceptibility to vaginal infections [12, 13, 16, 18]. In this context, the nominal association between *Gardnerella* abundance and FBG is noteworthy. FBG reflects basal hepatic glucose production and insulin resistance, whereas post-load glucose values capture dynamic insulin responses [36, 37]. The lack of association with 1 h and 2 h glucose may therefore indicate a closer link between vaginal microbial composition and underlying metabolic status rather than with short-term glycaemic fluctuations. Although causality cannot be inferred, these findings are compatible with the possibility that baseline metabolic disturbances, including insulin resistance-associated inflammation or immune dysregulation, may contribute to vaginal microbial shifts in women with GDM.

Mechanistically, metabolic stress in women with GDM may impair mucosal immunity and alter epithelial turnover, glycan availability and local immune surveillance, favouring expansion of non-*Lactobacillus* pathobionts [10]. *Gardnerella* can form biofilms, conferring a competitive advantage when *Lactobacillus*-mediated colonisation resistance is weakened [10, 38]. In parallel, insulin resistance-associated low-grade inflammation has been shown to reshape gut microbial communities [39], and the enrichment of *Gardnerella* observed here may represent a local manifestation of systemic metabolic stress within the vaginal ecosystem in women with GDM.

Beyond maternal glucose levels, the nominal association between *Gardnerella* abundance and fetal growth is of interest. Although GDM is typically linked to accelerated fetal growth [20], the inverse association observed within women with GDM suggests a possible growth-restrictive effect in a subset of such women. This pattern was absent in control participants and was weakened after excluding extreme values, warranting cautious interpretation. Rafat et al similarly reported higher BV prevalence in third-trimester GDM and associations with low birthweight, supporting the biological plausibility of a potential association between vaginal dysbiosis and lower birthweight in women with GDM [12]. Importantly, both *Gardnerella* abundance and fetal growth may reflect shared upstream metabolic or inflammatory processes that are not fully captured in this study. GDM-related disturbances extend beyond glucose metabolism to include dyslipidaemia and broader metabolic dysfunction, which may influence both *Gardnerella* enrichment and fetal growth trajectories [40–42]. Whether these associations are driven by unmeasured confounders requires further investigation.

Vaginal dysbiosis, particularly enrichment of *Gardnerella*, has been linked to proinflammatory cytokine profiles and low-grade inflammation at the maternal–fetal interface, with potential implications for placental vascular function and placental nutrient exchange [2, 43, 44]. *Gardnerella*-associated biofilm formation and mucosal disruption may further increase susceptibility to ascending infection [30, 45]. However, as participants with overt obstetric complications were excluded, the extent to which these mechanisms influence placental function and fetal growth in women with GDM remains unclear.

Functional predictions based on PICRUSt2 suggested that pathways related to the ‘nervous system’ category, including GABAergic and glutamatergic synapse-related terms, displayed directional correlations with *Gardnerella*, maternal FBG and fetal growth. Although these inferences do not represent direct measurements of microbial metabolic activity, their consistency with experimental evidence linking metabolic and neuroactive pathways to offspring outcomes suggests that future integrative metabolomic studies could explore the biological relevance of these pathways [21, 46].

Strengths of this study include the focus on women with diet-controlled mild GDM to minimise confounding from metabolic or obstetric complications, the use of standardised birthweight percentiles to reduce gestational age heterogeneity, and the integrated exploration of associations among maternal glucose levels, vaginal microbiota and fetal development. These findings provide a focused framework for future integrative studies combining metagenomics, metabolomics and placental functional assessments to elucidate microbiome-mediated mechanisms in women with GDM.

Several limitations should be acknowledged. Statistical power was limited in subgroup and correlation analyses, and the findings require confirmation in larger multicentre cohorts. The cross-sectional design precludes causal inference and limits assessment of temporal dynamics across pregnancy. OTU-based analysis may not capture strain-level variation, and higher-resolution approaches such as analysis of amplicon sequence variants are warranted in larger cohorts. Functional analyses were based on inferred pathways rather than directly measured metabolic activities, and should be considered hypothesis-generating. Finally, the absence of placental, cervical mucus or amniotic fluid samples limits direct assessment of ascending microbial exposure and inflammatory mechanisms.

Existing data on the vaginal microbiome in women with GDM remain limited and inconsistent, probably due to small sample size and confounding obstetric comorbidities [13, 16–18]. The structural shift observed in women with GDM, characterised by reduced *Lactobacillus* and increased *Gardnerella* abundance without increased overall diversity, aligns with findings in late pregnancy among women with type 1 diabetes, suggesting that metabolic dysregulation may reshape vaginal ecology within a low-diversity, host-modulated niche [47]. Our findings extend these observations to women with GDM, and specifically highlight *Gardnerella* as a genus of interest.

There are limited studies examining the relationship between the vaginal microbiota and fetal growth in women with GDM. The nominal association that we identified between *Gardnerella* and lower fetal growth is consistent with findings by Rafat et al, who reported a higher rate of BV in third-trimester GDM and its association with low birthweight [12]. In contrast to studies that have linked GDM mainly with increased risk of being large for gestational age (LGA) [19, 20], our findings suggest that the vaginal microbiota may contribute to heterogeneity in fetal growth within women with GDM, with some pregnancies showing potential growth restriction. Given the dichotomisation of birthweight percentiles (<50% vs ≥50%) and limited sample size, a more detailed classification (e.g. SGA and LGA) was not feasible; thus, these findings should be considered exploratory and hypothesis-generating.

Sex-stratified analyses indicated that nominal associations of *Gardnerella* abundance with FBW%ile and FBG were mainly observed in male fetuses. However, no significant interaction by fetal sex was detected and statistical power was limited. These findings should be interpreted cautiously, as extreme *Gardnerella* abundance was largely observed in women carrying male fetuses. Male fetuses are more vulnerable to intrauterine stress and have reduced placental adaptive capacity. Cidade-Rodrigues et al observed that, among the offspring of women with GDM, male infants, while presenting a higher mean birthweight, also exhibited an elevated SGA risk, highlighting the marked growth heterogeneity in this subgroup [22]. Our findings are consistent with, but do not establish, a microbiota-related contribution to fetal growth heterogeneity in male fetuses. Confirmation in adequately powered cohorts is needed.

The findings suggest that variation in vaginal microbial composition is associated with the heterogeneity in fetal growth observed among pregnancies affected by GDM, potentially serving as a downstream correlate of maternal metabolic status, or representing a parallel pathway alongside hyperglycaemia-driven anabolic effects. These findings have clinical implications, warranting further investigation into whether routine screening for vaginal dysbiosis or targeted microbial interventions could improve adverse pregnancy outcomes among women with GDM.

Several unanswered questions remain. Longitudinal studies are needed to establish temporal directionality between metabolic dysregulation, vaginal microbiome shifts and fetal growth trajectories. Higher-resolution metagenomic and metabolomic approaches are required to identify strain-specific features and microbial functional activities that may underlie the observed associations. The potential confounding role of maternal lipid metabolism should also be addressed in future studies that include comprehensive metabolic phenotyping.

### Conclusion

Late-pregnancy GDM was associated with modest alterations in vaginal microbial structure, and exploratory associations between *Gardnerella*, maternal fasting glucose and fetal growth were identified. The consistency of direction suggests a potentially relevant interface between maternal metabolic status, reproductive tract microbiota and fetal development in pregnancies affected by GDM. These findings may be of interest for future studies aiming to identify GDM-affected pregnancies at risk of relatively lower fetal growth, and to explore microbiome-targeted prevention or intervention strategies.

## Supplementary Information

Below is the link to the electronic supplementary material.

ESM (PDF 2569 KB)

## Data Availability

The raw sequence data reported in this paper have been deposited in the Genome Sequence Archive [48] of the National Genomics Data Center [49] (GSA: CRA033972), and are publicly accessible at https://ngdc.cncb.ac.cn/gsa.

## Abbreviations

ANOSIM: Analysis of similarities
BV: Bacterial vaginosis
COG: Clusters of Orthologous Groups (database)
FBHC%ile: Fetal birth head circumference percentile
FBW%ile: Fetal birthweight percentile
FDR: False discovery rate
GAS: Gestational age at sampling
GDM: Gestational diabetes mellitus
KEGG: Kyoto Encyclopedia of Genes and Genomes
LGA: Large for gestational age
OGTT: Oral glucose tolerance test
OTU: Operational taxonomic unit
PCoA: Principal coordinate analysis
SGA: Small for gestational age
VVC: Vulvovaginal candidiasis
