# Supplementary material for: Gardnerella enrichment in the vaginal microbiome of women with gestational diabetes mellitus is associated with lower fetal birthweight percentiles
Source: Diabetologia. 2026 Jun 11;69(9):2472–86. doi: 10.1007/s00125-026-06770-x (PMC13424189; doi:10.1007/s00125-026-06770-x)
Supplement: Supplementary file 1 — ESM (PDF 2569 KB) [file 125_2026_6770_MOESM1_ESM.pdf]

ESM Table 1 The characteristics and pregnancy outcomes for women with male and female fetuses' group in GDM

Characteristics and outcomes	Female fetuses' group (*n*=28)	Male fetuses' group (*n*=32)	*p* value
Maternal age (years)	30.4±3.0	31.8±3.7	0.111[a]
Pre-pregnancy BMI (kg/m^2)	20.8 (19.5–22.3)	22.1 (19.3–24.8)	0.382[b]
Gravidity			0.279[c]
1	22 (78.6)	19 (59.4)	
2	4 (14.3)	9 (28.1)	
>2	2 (7.1)	4 (12.5)	
Gestational age at sampling (weeks)	36.5±1.0	36.8±1.0	0.195[a]
FBG	4.7±0.5	4.8±0.5	0.422[a]
1 h blood glucose	10.7±1.4	10.3±1.5	0.441[a]
2 h blood glucose	9.0 (8.0–10.2)	8.7 (7.8–9.3)	0.156[b]
Gestational age at delivery (weeks)	39.2±0.8	38.7±0.8	0.013[a]
Cesarean delivery	13 (46.4)	25 (78.1)	0.011[c]
Birthweigth (g)	3095 (2968–3345)	3225 (3000–3443)	0.273[b]
FBW%ile	43.4±25.6	58.7±25.6	0.025[a]
FBHC%ile	62.7 (43.0–83.4)	78.4 (54.8–90.2)	0.159[b]
Apgar score at 1 min	8.0 (8.0–8.0)	8.0 (8.0–8.0)	0.350[b]
Apgar score at 5 min	9.0 (9.0–9.0)	9.0 (9.0–9.0)	1.000[b]
SGA	2 (7.1)	1 (3.1)	0.476[c]
LGA	1 (3.6)	4 (12.5)	0.212[c]

Values for continuous variables are presented as means ± SD or medians (IQR); values for categorical variables are expressed as *n* (%)

[a]Using Student's *t* tests

[b]Using the Wilcoxon rank-sum test

[c]Using the χ^2 test

ESM Fig. 1

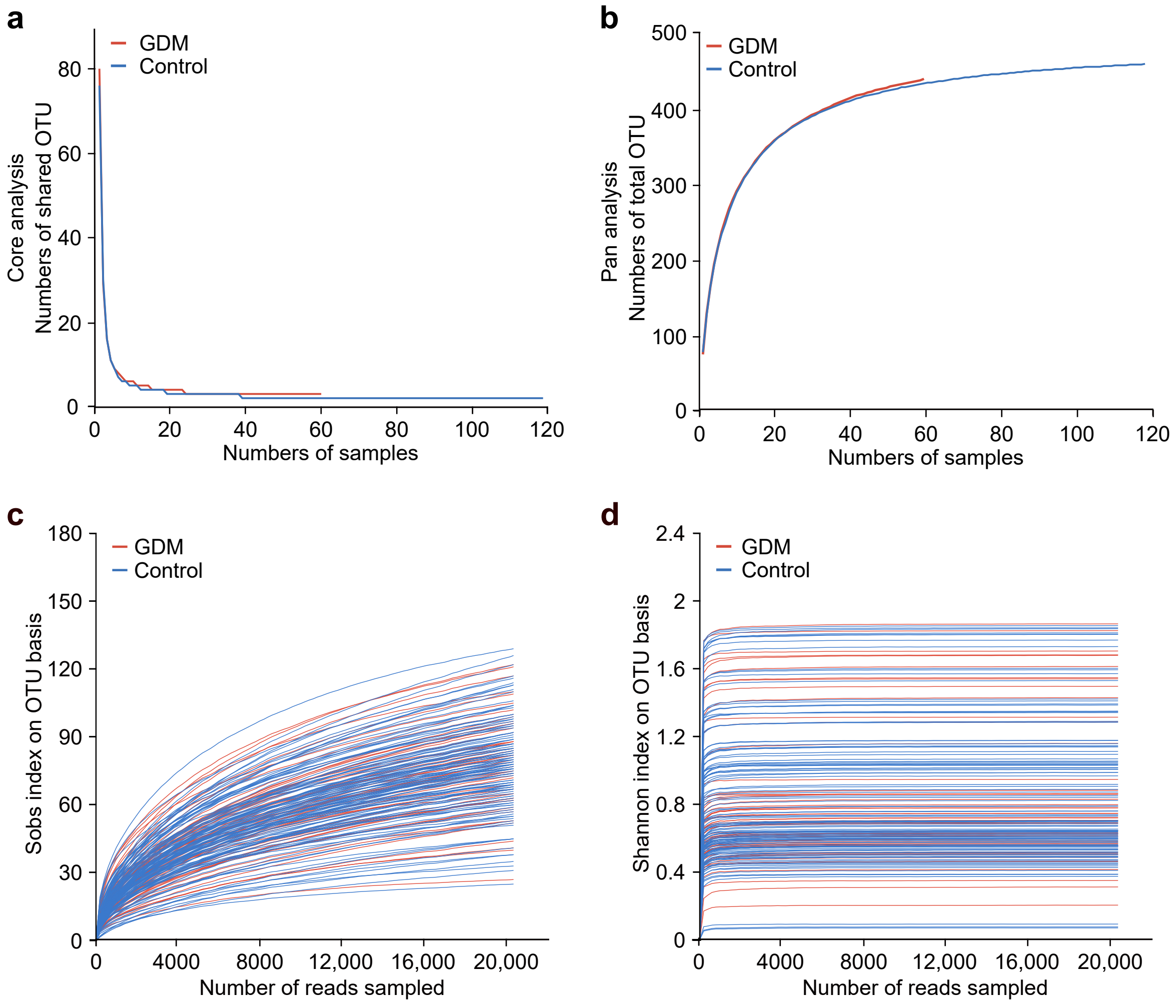

ESM Fig. 1 Sequencing depth was sufficient to capture vaginal microbiota diversity in both GDM and control groups. (a) Core microbiome accumulation curves showing the number of shared OTUs as a function of sample number in GDM and control groups, approaching saturation with increasing samples. (b) Pan microbiome accumulation curves showing the cumulative number of observed OTUs with increasing sample number, demonstrating comparable richness capture between groups. (c, d) Rarefaction curves showing stabilization of richness (Sobs index) and community diversity (Shannon index) with increasing sequencing depth.

ESM Fig. 2

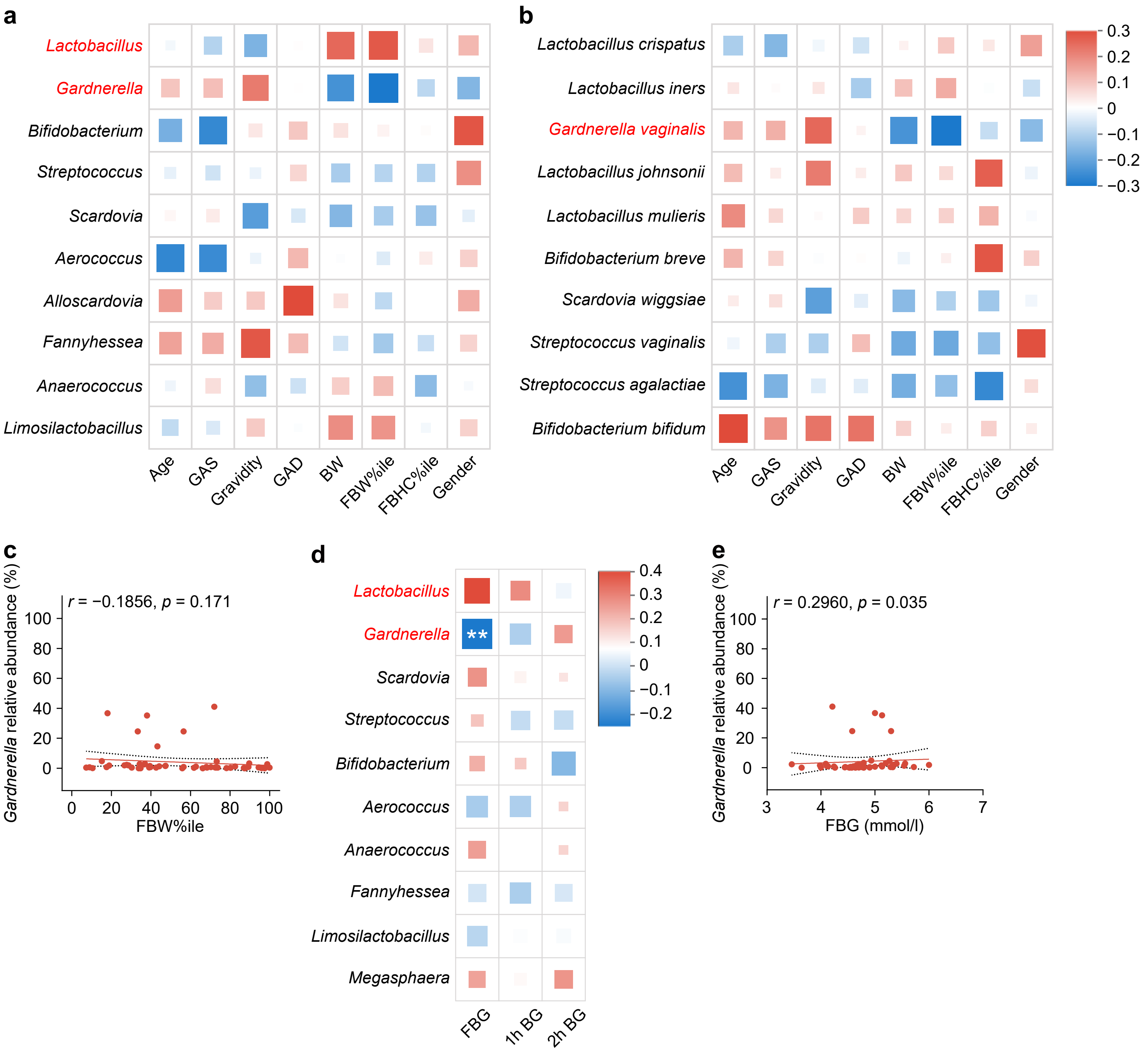

ESM Fig. 2 Associations between vaginal bacterial taxa and clinical variables. (a) Heatmap showing correlations between selected genera and clinical variables, including age, gestational age at sampling (GAS), Gravidity, gestational age at delivery (GAD) and fetal growth indices. For multiple comparisons, the Benjamini–Hochberg method was applied, with FDR<0.20 considered nominally suggestive; * represented *p*<0.05 and ** represented *p*<0.01 based on Spearman's rank correlation. (b) Heatmap showing corresponding associations at the species level. Colour scales indicate the direction and magnitude of correlations. For multiple comparisons, the Benjamini–Hochberg method was applied, with FDR<0.20 considered nominally suggestive; * represented *p*<0.05 and ** represented *p*<0.01 based on Spearman's rank correlation. (c) Sensitivity analysis after excluding outliers. Scatter plots showing associations between *Gardnerella* relative abundance and fetal birthweight percentile (FBW%ile), with fitted regression lines and 95% CIs. (d) Heatmap showing associations between top 10 genera and OGTT indices, including fasting blood glucose (FBG) and OGTT 1h and 2h blood glucose (BG). For multiple comparisons, the Benjamini–Hochberg method was applied, with FDR<0.20 considered nominally suggestive; * represented *p*<0.05 and ** represented *p*<0.01 based on Spearman's rank correlation. (e) Sensitivity analysis after excluding outliers. Scatter plots showing associations between *Gardnerella* relative abundance and FBG, with fitted regression lines and 95% CIs.

ESM Fig. 3

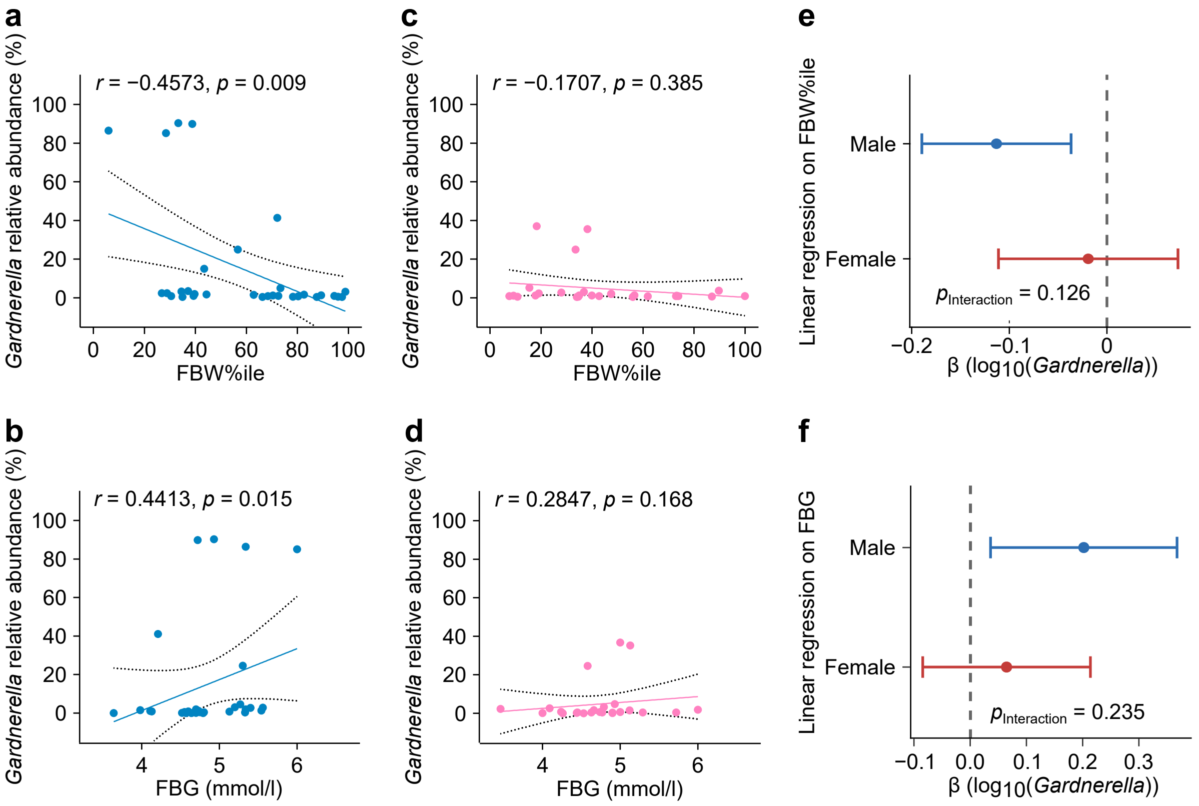

ESM Fig. 3 Sex-stratified associations between *Gardnerella* relative abundance and clinical variables. (a, b) Scatter plots showing associations between fetal birthweight percentile (FBW%ile),fasting blood glucose (FBG) and *Gardnerella* relative abundance in the male fetuses' groups. Associations were assessed using Spearman's rank correlation. (c, d) Scatter plots showing associations between FBW%ile, FBG and *Gardnerella* relative abundance in the female fetuses' groups. Associations were assessed using Spearman's rank correlation. (e, f) Forest plot showing subgroup analyses of the association between FBW%ile, FBG and *Gardnerella* abundance stratified by fetal sex, based on linear regression models with interaction testing. Solid lines indicate fitted regression lines and dotted lines indicate 95% CIs.

ESM Fig. 4

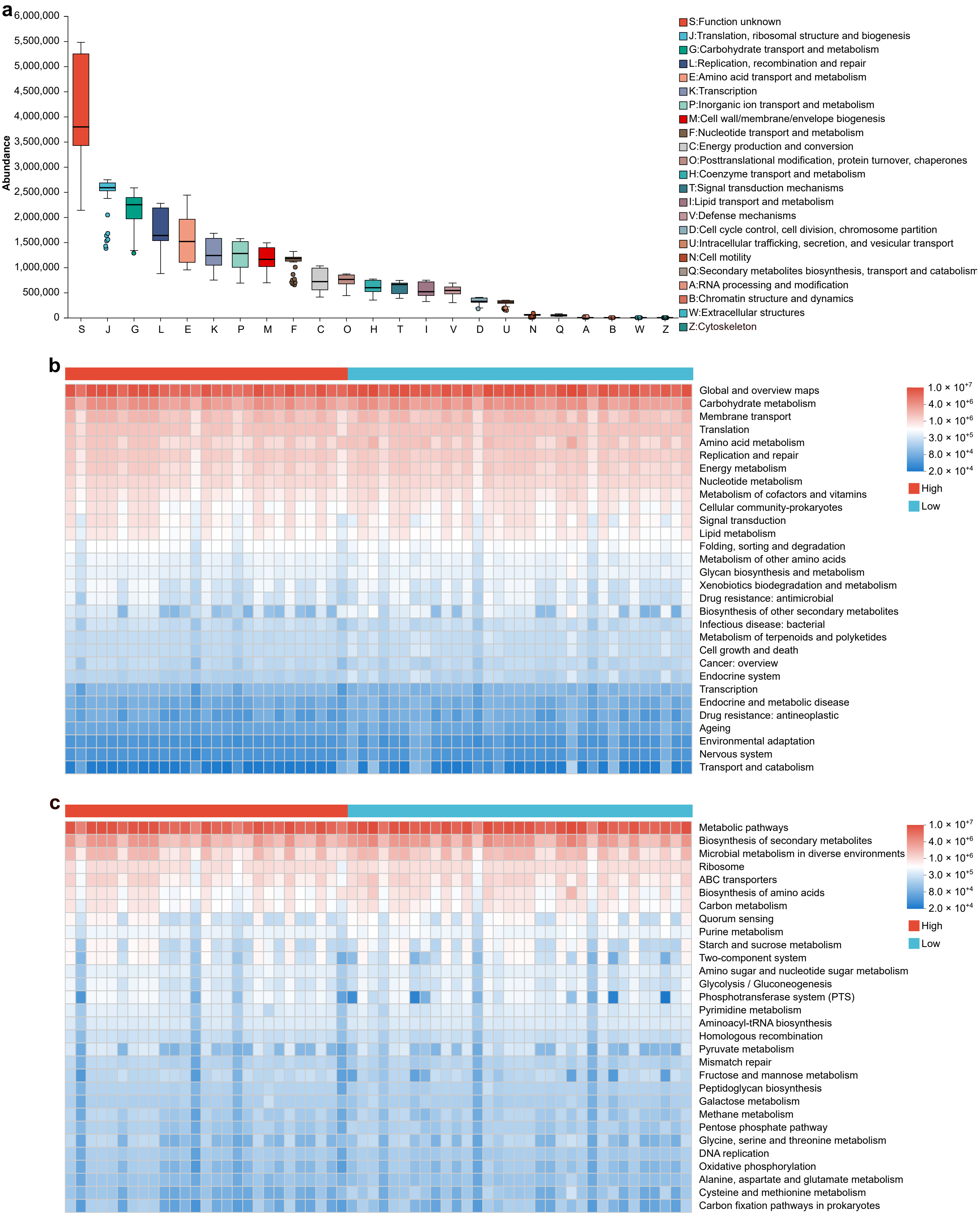

ESM Fig. 3 Predicted functional profiles of the vaginal microbiota inferred from 16S rRNA gene data. (a) Distribution of predicted functional categories based on COG classification using PICRUSt2. (b) Heatmap of predicted KEGG pathway level 2 functions across samples. (c) Heatmap of predicted KEGG pathway level 3 functions, showing finer-scale functional annotations. Functional prediction was performed using PICRUSt2 based on 16S rRNA gene profiles.

ESM Fig. 5

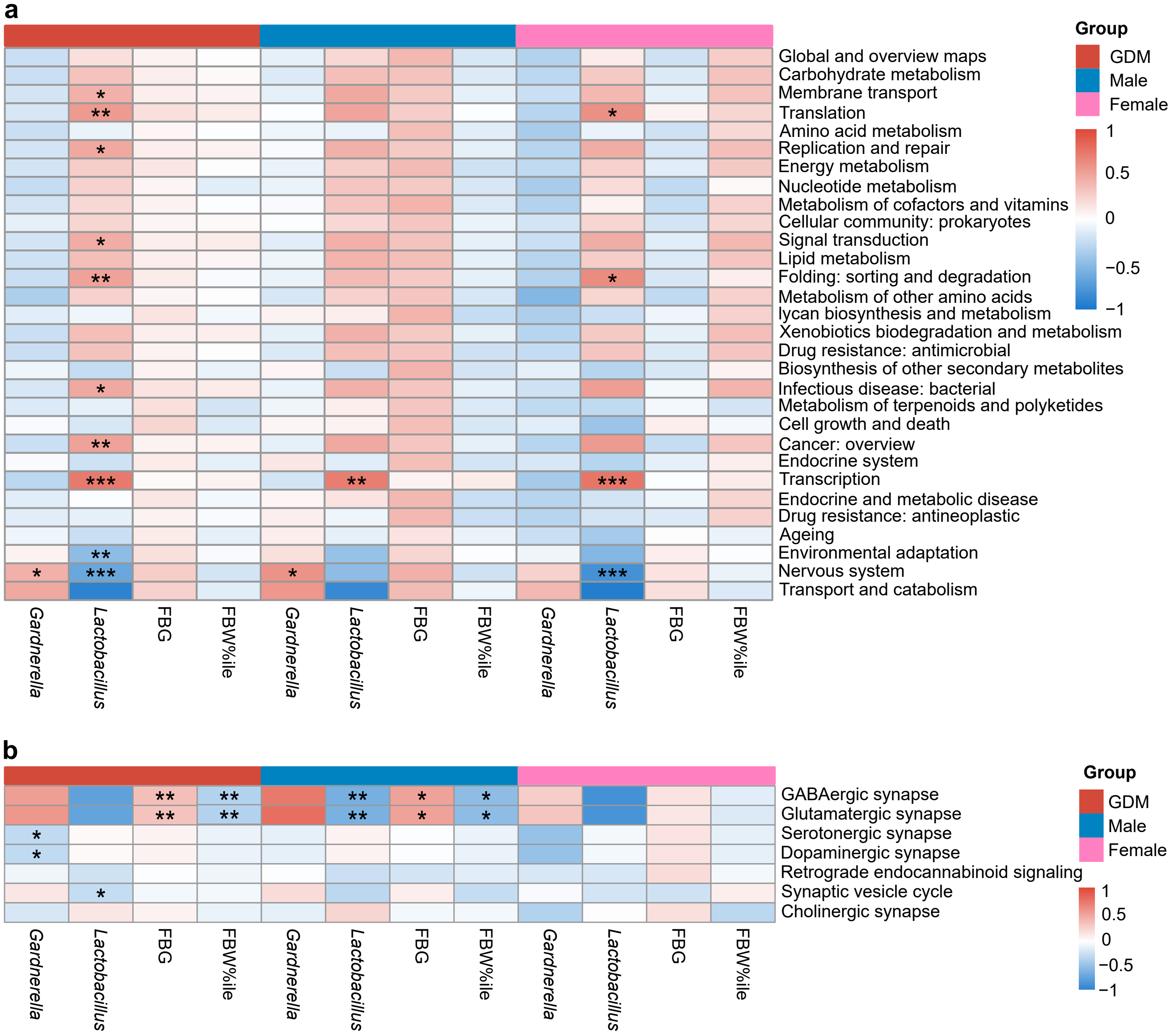

ESM Fig. 5 Associations between predicted microbial functional pathways and clinical variables. (a) Sensitivity analysis after excluding outliers. Heatmap showing correlations between selected KEGG pathway level 2 functions and clinical variables, including bacterial genera, fasting blood glucose and fetal birthweight percentile, across GDM, male and female fetuses' groups based on Spearman's rank correlation. * represented *q*FDR <0.05,** represented *q*FDR <0.01 and *** represented *q*FDR <0.001 based on Benjamini–Hochberg method. (b) Heatmap showing correlations between selected KEGG pathway level 3 functions and the same variables based on Spearman's rank correlation. Colour scales indicate the direction and magnitude of associations. Functional profiles were predicted using PICRUSt2 based on 16S rRNA gene data. * represented *q*FDR <0.05,** represented *q*FDR <0.01 and *** represented *q*FDR <0.001 based on Benjamini–Hochberg method.
